# Assessment of Phenanthrene Degradation Potential by Plant-Growth-Promoting Endophytic Strain *Pseudomonas chlororaphis* 23aP Isolated from *Chamaecytisus albus* (Hacq.) Rothm.

**DOI:** 10.3390/molecules28227581

**Published:** 2023-11-14

**Authors:** Magdalena Anna Karaś, Sylwia Wdowiak-Wróbel, Monika Marek-Kozaczuk, Wojciech Sokołowski, Krystsina Melianchuk, Iwona Komaniecka

**Affiliations:** Department of Genetics and Microbiology, Institute of Biological Sciences, Faculty of Biology and Biotechnology, Maria Curie-Skłodowska University, Akademicka 19, 20-033 Lublin, Poland; sylwia.wdowiak-wrobel@mail.umcs.pl (S.W.-W.); monika.marek-kozaczuk@mail.umcs.pl (M.M.-K.); wojciech.sokolowski@mail.umcs.pl (W.S.);

**Keywords:** *Pseudomonas chlororaphis*, biodegradation, phenanthrene, plant-growth-promoting (PGP) activities, biosurfactants, 2,6-dichlorophenolindophenol (DCPIP), GC-MS, DI-ESI-MS

## Abstract

Polycyclic aromatic hydrocarbons (PAHs) are common xenobiotics that are detrimental to the environment and human health. Bacterial endophytes, having the capacity to degrade PAHs, and plant growth promotion (PGP) may facilitate their biodegradation. In this study, phenanthrene (PHE) utilization of a newly isolated PGP endophytic strain of *Pseudomonas chlororaphis* 23aP and factors affecting the process were evaluated. The data obtained showed that strain 23aP utilized PHE in a wide range of concentrations (6–100 ppm). Ethyl-acetate-extractable metabolites obtained from the PHE-enriched cultures were analyzed by gas chromatography–mass spectrometry (GC-MS) and thin-layer chromatography (HPTLC). The analysis identified phthalic acid, 3-(1-naphthyl)allyl alcohol, 2-hydroxybenzalpyruvic acid, *α*-naphthol, and 2-phenylbenzaldehyde, and allowed us to propose that the PHE degradation pathway of strain 23aP is initiated at the 1,2-, 3,4-carbon positions, while the 9,10-C pathway starts with non-enzymatic oxidation and is continued by the downstream phthalic pathway. Moreover, the production of the biosurfactants, mono- (Rha-C_8_-C_8_, Rha-C_10_-C_8:1_, Rha-C_12:2_-C_10_, and Rha-C_12:1_-C_12:1_) and dirhamnolipids (Rha-Rha-C_8_-C_10_), was confirmed using direct injection–electrospray ionization–mass spectrometry (DI-ESI-MS) technique. Changes in the bacterial surface cell properties in the presence of PHE of increased hydrophobicity were assessed with the microbial adhesion to hydrocarbons (MATH) assay. Altogether, this suggests the strain 23aP might be used in bioaugmentation—a biological method supporting the removal of pollutants from contaminated environments.

## 1. Introduction

Polycyclic aromatic hydrocarbons (PAH) are persistent organic pollutants (POPs) of anthropogenic and natural origin, ubiquitous in the environment, and pose a public health hazard. Among them, those having four or more rings have been determined to be highly teratogenic, mutagenic, and carcinogenic to microorganisms, plants, and animals. While low-molecular-weight PAHs with three or less rings have not been confirmed as mutagenic and carcinogenic agents in humans, they negatively affect living organisms as toxic, irritating, and allergenic compounds. Since phenanthrene (PHE), consisting of three condensed benzene rings, is the smallest aromatic hydrocarbon to have a “bay-region”, and a “K-region” that can form highly reactive epoxides and is mainly responsible for the toxicity of carcinogenic PAHs, it is often used as a model substrate for studying their biodegradation [1].

Among the various methods of POP removal, the environmentally friendly method of using the participation of microorganisms seems to be the most promising solution [2], especially with the use of Gram-negative bacteria—classified in the genus *Pseudomonas* and having a very flexible genome—which translates into their capabilities to degrade and metabolize various xenobiotics [3]. Particularly valuable are the non-pathogenic strains belonging to the subgroup *Pseudomonas chlororaphis*, which have a range of positive agricultural characteristics. They are able to synthesize various molecules with antimicrobial or plant-growth-promoting (PGP) activities [4]. Also, they participate in the biodegradation of organic pollutants [5,6,7].

Some strains of *Pseudomonas* can use phenanthrene as the only source of carbon and energy, and the others can through co-metabolism [8,9,10]. The degradation process is preceded by the activation of stable aromatic rings by hydrogenation. Then, oxidized PHE undergoes further transformations, leading to a reduction in the number of rings and an increase in the polarity. The process is initiated by mono- or dioxygenases, which introduce an oxygen atom into two atoms of the benzene ring, resulting in the formation of *cis*-dihydrodiols. In the case of Gram-negative bacteria, dioxygenation most frequently concerns carbons in the 3,4-position, then 1,2-, and much less 9,10- in the phenanthrene rings with the formation of the corresponding diols. 3,4- or 1,2-*cis*-dihydrodiols are further oxidized to 1-hydroxy-2-naphthoic acid (1H2NA), which is directed to the peripheral “*ortho*” or “*meta*” cleavage pathways (phthalic acid or salicylic acid/naphthalene pathways, respectively). A type of ring fission termed *ortho*-cleavage occurs between the hydroxyl groups (intradiol cleavage) and *meta*-cleavage takes place when it occurs adjacent to one of the hydroxyls (extradiol cleavage). In addition, there is an alternative route that metabolizes 1H2NA via *α*-naphthol, which is then broken down to salicylates or phthalates. In turn, the ring opening of 9,10-dihydrodiol predominantly occurs via *ortho*-cleavage and leads mostly to the formation of diphenic acid as the end product [11].

In recent years, numerous studies on the degradation of PHE with the participation of various bacteria, including *Pseudomonas* strains, were published. However, plant–microbe systems, which utilize microbe-assisted phytoremediation, seem to exhibit a better potential for the removal of POPs due to the mutually beneficial behaviors of participants causing an increase in the remediation efficiency. Plants can provide nutrients to support the growth and proliferation of microorganisms, increase their activity, shift the microbial community structure, and alter microbial gene expression. In return, microbes can improve the availability of nitrogen, phosphorus, and potassium to the plants; release exogenous somatotrophic hormones; show antagonistic interactions with pathogenic bacteria; and degrade compounds that are toxic to plants [12]. So far, such interactions were mostly considered in relation to rhizosphere bacteria [13]. However, since endophytes develop more stable interactions with plants and can be more useful in the considered technique of bioremediation, increasing attention is being paid to this topic [14,15,16,17].

Thus, the aim of this study is to (i) isolate the endophytic strain capable of PHE utilization as well as evaluate factors affecting the process; (ii) determine the taxonomic position of the studied strain; (iii) assess its plant-growth-promoting activities (PGP); (iv) identify the metabolites from PHE degradation with the used strain and, on the basis of this, propose the pathways of its degradation; and, finally, (v) to discover other characteristics that increase phenanthrene bioavailability with the participation of the chosen isolate.

## 2. Results

### 2.1. Isolation and Identification of the PHE-Degrading-Strain with PGP Activities

The study analyzed endophytic strains isolated from *Chamaecytisus albus* nodules. The screening assays performed on mineral salt agar medium (MSM) sprayed with a PHE solution showed that four Gram-negative rod-shaped bacteria had the potential to degrade the tested hydrocarbon. The possibility of utilizing phenanthrene as a sole source of carbon and energy was evidenced by the appearance of clear (transparent) zones surrounding the colonies in enriched agar cultures (Figure S1). One of these strains (23aP), showing the best tolerance and ability to acclimatize to PHE in the range of 25–500 ppm and exhibiting the highest viability confirmed by the CFU determination, was selected for further research. Strain 23aP was also able to utilize fluorene and acenaphthene but not anthracene.

To assess the phylogenetic position of strain 23aP, a multilocus sequence analysis (MLSA) of combined partial sequences of three housekeeping genes (*recA*, *gyrB*, *rpoB*) with a 1735 bp length of the isolated strain was performed for comparison with the corresponding gene sequences of the reference strains available in the GenBank database.

**Figure 1:**
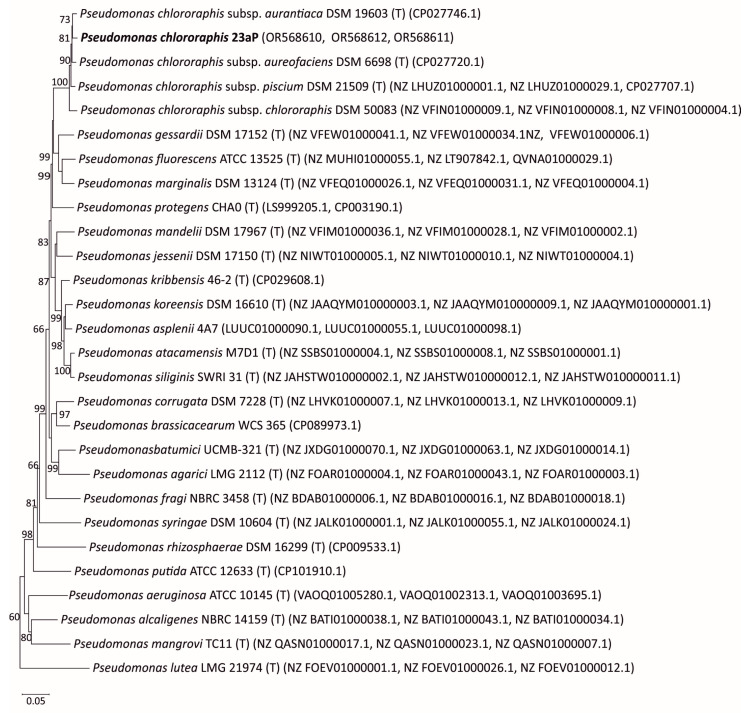
Phylogenetic tree based on the three-gene concatenated sequences (*gyrB*, *rpoB*, *recA*) (Neighbor-Joining method; NJ). Bootstrap values ≥ 50 are indicated in the nodes. Bar indicates sequence divergence.

The MLSA analysis showed that strain 23aP had the highest degree of sequence similarity to species belonging to the *Pseudomonas* genus (sequence similarity 80–98%). The phylogenetic analysis included 27 reference strains representing 24 species of the *Pseudomonas* genus (Figure 1). The 23aP strain formed a group with strains of the *Pseudomonas chlororaphis* (23aP) species (with a support rate of 100%). The sequence similarity of the tested strain to the sequence of *P. chlororaphis* strains ranged from 97% to 98%. The most closely related strain to 23aP was *P. chlororaphis* subsp. *auriantiaca* DSM19603 (sequence similarity 98%).

The endophytic *P. chlororaphis* 23aP strain was isolated from root nodules of a legume plant; thus, it was expected to have PGP attributes [18]. As per expectation, the isolate exhibited both direct and indirect plant-growth-promoting traits. The evaluation of the quantity of siderophore and hydrogen cyanide produced by the tested strain indicated its capacity to produce these antimicrobial compounds. Moreover, strain 23aP was effective in dissolving phosphates; it exhibited proteolytic and cellulolytic activity but did not produce IAA (idole-3-acetic acid) (Table S1).

### 2.2. Factors Affecting the Degradation of Phenanthrene

The oxidation of PHE by microorganisms involves redox reactions, in which electrons are transferred to electron acceptors. The first step in the biodegradation of PHE consisting of ring-hydroxylation is initiated by NADH-dependent oxidoreductases, i.e., multicomponent enzyme systems involving several proteins and nonheme iron as dioxygenases or monooxygenases [11]. To assess the level of PHE degradation, it is possible to use the 2,6-dichlorophenolindophenol (DCPIP) assay. In this method, during enzyme-catalyzed oxidation, the synthetic DCPIP mediator accepts electrons from NAD(H)P, which is observed as a change in its color from blue (oxidized form) into the colorless reduced form. The reduction activity is a sensitive indicator directly coupled to respiration via the electron transport chain and, thus, reflects the metabolic status of the cell. Microorganisms that are able to use PHE as a source of carbon and energy are metabolically active. Consequently, the utilization of PHE is confirmed indirectly by an increase in bacterial activity observed as a decrease in absorbance (*A*) at a wavelength of 600 nm, which is a maximum of absorption for oxidized DCPIP [19].

Since the growth of bacteria depends on various environmental factors, which may affect the effectiveness of PHE utilization, the culture of strain 23aP was optimized in terms of selected physicochemical conditions using single-component experiments. In studies on the optimization of bacterial cultures, changes in biomass over time are most often examined using spectrophotometric methods. This quantity is expressed as optical density (OD). In the present study, the OD measurements turned out to be impossible due to the aggregation of bacteria and changes in their size in the presence of PHE, which resulted in a decrease in OD over time with a simultaneous increase in CFU. Therefore, the capability of PHE degradation by strain 23aP was tested using the DCPIP assay.

Prior to the current study, the basic culture conditions of the isolated 23aP strain in MSM medium were established. Thus, the following parameters were selected for evaluation of PHE degradation efficiency using the DCPIP assay: the rate of agitation, nitrogen sources, and the initial PHE concentration.

**Figure 2:**
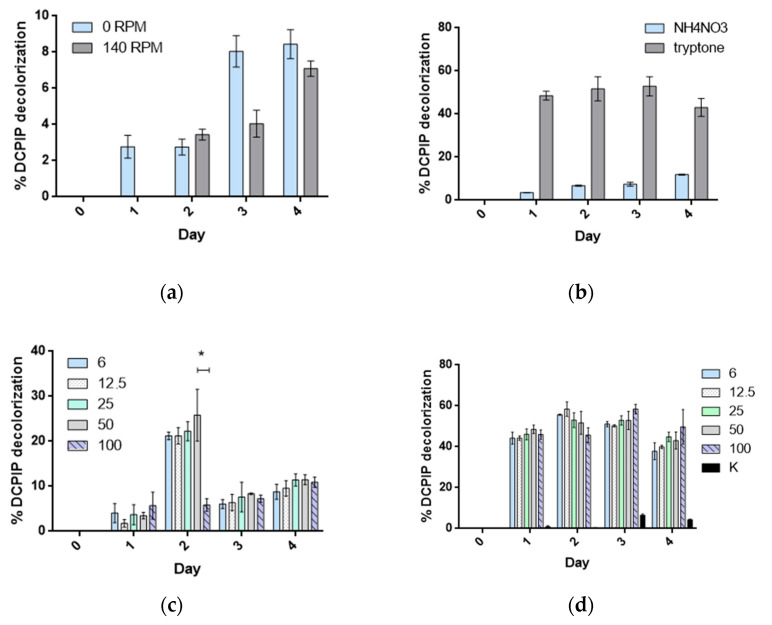
Phenanthrene degradation by *Pseudomonas chlororaphis* strain 23aP in DCPIP assay involving variable (**a**) rpm, (**b**) nitrogen sources, (**c**) PHE concentration in the presence of ammonium nitrate (0.1%), and (**d**) PHE concentration in the presence of tryptone (0.1%); * *p* < 0.0001, K—abiotic control.

The comparison of the growth profiles (Figure 2a) indicated that the yield of PHE utilization should be higher in stationary cultures of strain 23aP (*p* < 0.05). Therefore, cultures without agitation with two representative sources of nitrogen were carried out in this study. Their results showed that the kinetics of strain 23aP growth with PHE as a carbon source was higher in the presence of tryptone (*p* < 0.0001) than when the cultures were supplemented with ammonium nitrate (Figure 2b). In the MSM cultures supplemented with tryptone, there were no significant differences in the growth rate and abundance of strain 23aP, regardless of the initial PHE concentration during the first three days of the experiment. However, careful inspection of the profiles revealed that, in the presence of 25, 50, and 100 ppm of PHE, strain 23aP showed diauxic growth. This suggests that tryptone is utilized by *P. chlororaphis* 23aP not only as a nitrogen source but also as a source of carbon, which was further confirmed in cultures on the MSM/tryptone medium without other C sources. In the case of cultures with ammonium nitrate, the highest kinetics of strain 23aP growth was obtained when 50 ppm of PHE was added to the medium.

### 2.3. Phenanthrene Metabolic Intermediates

Despite the indications that strain 23aP utilized PHE with a higher yield when the MSM medium was supplemented with tryptone, cultures enriched with ammonium nitrate as a nitrogen source were used to obtain intermediate products of phenanthrene degradation, since we established that PHE might be degraded through co-metabolism in the presence of tryptone. Static cultures containing 50 ppm PHE were run for 2 and 7 days. At the specified time points, metabolites were extracted from cell-free supernatants with ethyl acetate. Then, combined neutral and acidic extracts were analyzed with GC-MS.

In this study, a total of 10 predicted PHE degradation products present in the culture fluids were identified using GC-MS. In chromatograms from a sample of the 48 h culture, peaks registered at 6.35, 11.22, 11.55, 13.94, and 15.96 min corresponded to *α*-naphthol, 9*H*-fluorene-9-one, 3-(1-naphthyl)allyl alcohol, 2-phenylbenzaldehyde, and 9,10-phenanthrenequinone, respectively. The annotations were made on the basis of the registered *m*/*z* values and by matching mass fragmentation patterns with the mass spectra in the NIST (National Institute of Standards and Technology) library and in the literature [20,21,22,23]. The fragmentation pattern of 3-(1-naphthyl)allyl alcohol was in line with previous data reported by Nzila et al. [24]. In addition to those mentioned above, several peaks with a fragment at *m*/*z* 149, which are characteristic of alkylated derivatives of phthalic acid, were identified as well (Table 1). The GC-MS analysis of the TMS derivatives from the same sample revealed additional compounds: one that eluted at 5.58 min corresponding to acetophenone according to the NIST library and another one eluted at 7.04 min, which consisted of the following MS fragments: *m*/*z* 278 (M^+^), 263 (M^+^ − CH_3_), 247 (M^+^ − OCH_3_), 191 (M^+^ − Si(CH_3_) from 263 ion), 175 ((M^+^ − OSi(CH_3_) from 263 ion), and 147 (M^+^ − COOCH_3_ from 191), similarly to the MS data reported previously for the TMS methyl ester derivative of 2-hydroxybenzalpyruvic acid [24]. The analysis of the 7 d culture sample showed that similar compounds, except for acetophenone, were present in the culture fluids as during the shorter period of incubation of 23aP with PHE. Some amounts of 9,10-phenanthrenequinone and 9*H*-fluorene-9-one were also found in the abiotic controls.

Most of the identified compounds are lower pathway intermediates generated from PHE with the participation of strain 23aP. No initial metabolic intermediates involved in the upper catabolic pathway were observed. In all the analyses, a compound that eluted at 12.133 min with the molecular ion (M^+^) at *m*/*z* 178 was identified as phenanthrene based on the retention time and fragmentation pattern of the authentic standard.

The HPTLC analysis of ethyl-acetate-extractable metabolites of phenanthrene in the hexan:chloroform:acetic acid (10:3:2, *v*/*v*/*v*) solvent system revealed six spots (Figure S2, Table S2) visible under short- and long-wave UV light. One of the components showing blue fluorescence (R_f_ 0.37) migrated similarly to the reference α-naphthol and salicylic acid, which had R_f_ values of 0.33 and 0.36, respectively. Since the literature data [25,26] indicate that such fluorescence is a feature of both salicylic acid and 1H2NA with a similar migration coefficient in this solvent system, another HPTLC analysis was performed in a mixture of chloroform:methanol:water (65:15:2, *v*/*v*/*v*). The obtained chromatogram (Figure S3, Table S3) showed that one of the metabolites migrated similarly to α-naphthol with R_f_ 0.67 as a commercial standard and, like this compound, did not show fluorescence. None of the compounds had the migration coefficient of the reference salicylic acid. In turn, the metabolite that showed blue fluorescence had R_f_ 0.77, possibly indicating that it represented 1H2NA, which was not detected in the GC-MS analyses.

### 2.4. Mechanisms of Increasing Phenanthrene Bioavailability

Since the degradation of PAHs takes place in the cytoplasm of bacterial cells and PAHs are highly hydrophobic compounds that strongly interact with organic matter in the soil, to increase their bioavailability, microorganisms produce substances called biosurfactants. These are amphiphilic molecules characterized by the ability to reduce surface tension at the phase boundary, thus increasing PAH bioavailability. Since some strains of *Pseudomonas* spp. mainly produce biosurfactants classified as anionic glycolipids called rhamnolipids (RhaL) [27], an attempt was made to obtain and analyze biosurfactants from the 23aP strain.

Rhamnolipids composed of one or two β-hydroxy fatty acid residues with different lengths (varying from C_8_ to C_16_) and saturations (saturated, mono-, or polyunsaturated) attached to one or two L-rhamnose moieties are a diverse group of molecules representing over 60 reported congeners [27]. Since different bacterial strains produce individual RhaL mixtures, the extraction method may affect the yield of recovery [28].

To overcome this limitation and to obtain the highest productivity of rhamnolipids from the strain 23aP fermentation broths for analyses, different aqueous two-phase extraction methods were employed (according to the Folch method—sample 1 and with ethyl acetate—sample 2). Obtained crude rhamnolipid preparations were applied to HPTLC (Figure S4), followed by characterization of the congener composition using DI-ESI-MS in the negative ionization mode. In order to confirm the structures and position of the fatty acids, the most abundant ions were submitted to the tandem-MS mode. Most of the congeners detected were attributed to mono-RhaLs, and only one homolog was assigned to di-RhaL. Altogether, five rhamnolipid homologs were identified (Table 2).

The ESI-MS analysis of sample 1 gave three pseudo-molecular ions at *m*/*z* 527, 555, and 621. In the tandem mass mode, ions *m*/*z* 527 (Rha-C_12:2_-C_10_) and 621 (Rha-Rha-C_8_-C_10_) gave daughter diagnostic ions at *m*/*z* 357 and 451 corresponding to the rupture of the ester link, while the ion at *m*/*z* 555 (Rha-C_12:1_-C_12:1_) gave a diagnostic ion at *m*/*z* 195 corresponding to the released fatty acid (C_12:1_). Sample 2, i.e., the ethyl-acetate extract, gave two pseudomolecular ions at *m*/*z* 447 and 473 assigned to mono-RhaLs. The ion with *m*/*z* 473 (Rha-C_10_-C_8:1_) gave a daughter ion at *m*/*z* 333 corresponding to Rha-C_10_. The ion at *m*/*z* 447 (Rha-C_8_-C_8_) was identified by an accurate mass assignment. Based on the relative intensity of the recorded peaks, it can be concluded that Rha-C_10_-C_8:1_ was produced in the greatest amount.

After the application of moderately polar ethyl acetate as an organic solvent, only mono-RhaLs were identified in the analyses. In turn, the use of the mixture of non-polar chloroform and polar methanol in the Folch method gave mono-RhaLs with FAs with longer aliphatic chains and, additionally, one class of di-RhaLs.

It is known that biosurfactants produced by bacteria can either occur on the cell surface or be secreted extracellularly. Bacteria can produce both groups of biosurfactants or only a selected one. A manifestation of the presence of rhamnolipids associated with bacterial cells is the increase in the hydrophobicity of their surface, which results from the substitution of lipopolysaccharide molecules with rhamnolipids in the outer membrane. The mechanism of this phenomenon was described in detail by Ma et al. [29].

To check whether this mechanism of increasing the PHE bioavailability is employed by strain 23aP, changes in the hydrophobicity of the bacterial cell surface in the presence of phenanthrene were assessed using the microbial adhesion to hydrocarbons (MATH) assay [30]. Bacteria grown in the presence of phenanthrene as a sole source of carbon and energy and in the presence of the selected glucose and tryptone co-substrates in the MATH test gave results of 7.21 ± 0.5, 30.19 ± 1.24, and 47.44 ± 1.83%, respectively. The results from the MATH assay indicate that 23aP cultured in the presence of PHE with tryptone as a co-metabolite had the highest cell surface hydrophobicity (*p* < 0.005), which provides the best conditions for hydrocarbon uptake from the environment and may have an impact on the rate of degradation. In turn, the low surface hydrophobicity of 23aP cultured with PHE as the only source of carbon and energy with a non-organic nitrogen source shows significantly lower efficiency of PHE uptake in such conditions, which results in a lower rate of PHE degradation. This confirms the findings from studies on the kinetics of 23aP growth using the DCPIP indicator and our observations of the number of PHE crystals remaining after 7 days of enriched cultures.

## 3. Discussion

Terrestrial ecosystems are subjected to high anthropogenic pressure that leads to the accumulation of toxic and recalcitrant POPs, which are prone to long-range atmospheric transboundary migration and deposition far from the sources of their emission. Thus, they pose a public health hazard and exert serious effects on wildlife and biota adjacent to and distant from their origin of emission [31]. Especially, human exposure to PAHs by inhalation, ingestion, and dermal contact can exert both short- (allergy, skin irritation, and inflammation) and long-term health consequences (mutagenic, carcinogenic, and teratogenic effects). However, their reactive biotransformation products, such as diol epoxides and dihydrodiols, are the key factors of the carcinogenic potential of PAHs [32].

Phenanthrene—a low-molecular-weight PAH widely distributed in the environment—is classified as category D for human carcinogenicity by the US Environmental Protection Agency, i.e., it is not classifiable as to human carcinogenicity. However, due to the similarity of its structure to higher PAHs with carcinogenic properties and the reported formation of its bioactive forms in the environment as a result of the action of reactive oxygen species, it is a model compound in research on PAH biodegradation [33].

Moreover, as common environmental contaminants, PAHs (including PHE) can have severe impacts on flora and fauna as immunotoxic agents, lowering reproduction in animals, inducing oxidative stress, and lowering seed germination and biomass production rates as well as the total chlorophyll content in plants, just to name a few [17,34]. Although a range of natural dissipation processes—e.g., volatilization, photooxidation, chemical oxidation, sorption, and leaching—can lower their content and, thus, reduce their ecotoxicity, biodegradation by microorganisms has been generally considered to be one of the primary means for their removal from the environment. Bacterial species having an ability to degrade phenanthrene via their utilization as a sole carbon and energy source include diazotrophic rhizobia and potential non-symbiotic endophytes of leguminous plant root nodules from the genera *Pseudomonas*, *Shingobium*, *Rhizobium*, *Sinorhizobium (Ensifer)*, *Mycobacterium*, *Burkholderia*, *Stenotrophomonas*, and *Sphingomonas* [10,11,22,35,36,37,38,39]. In this study, we have demonstrated that the endophytic *Pseudomonas chlororaphis* 23aP strain can utilize such versatile PAHs as phenanthrene, fluorene, and acenaphthene as the only sources of carbon and energy. Data from the PHE-enriched cultures with the redox indicator DCPIP showed that the optimal initial concentration of phenanthrene for its degradation by 23aP was 50 ppm, but the strain remained viable within the range of 25–500 ppm. This is consistent with data from previous reports indicating that bacteria are able to utilize phenanthrene in a wide range of concentrations from 1 to 1000 ppm, with the optimum usually at 25–500 ppm [24,40,41,42]. Currently, the optimal concentration of PHE has been established for cultures supplemented with ammonium nitrate since tryptone appeared to be used by strain 23aP not only as a nitrogen source but also as a carbon source. Besides, most studies on PHE degradation by strains classified to *Pseudomonas* were carried out with vigorous agitation [10,25], whereas the stationary cultures of strain 23aP contributed to better kinetics of growth in the presence of PHE.

**Figure 3:**
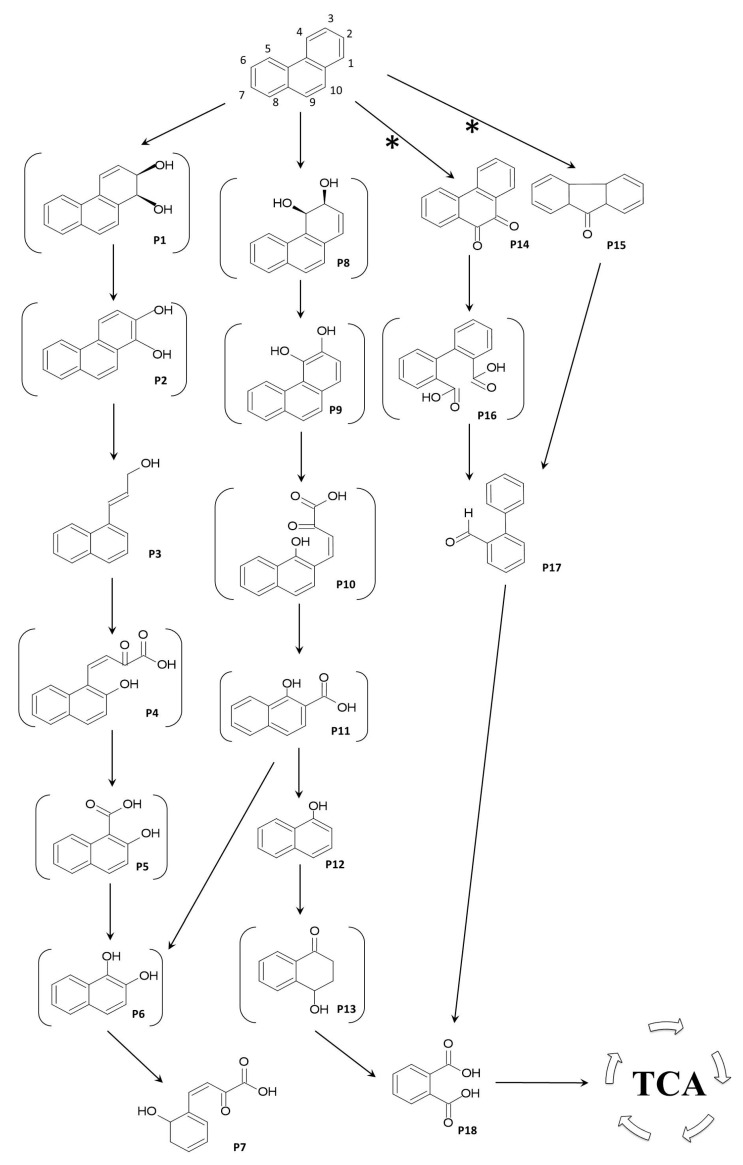
Proposed metabolic pathway for the degradation of phenanthrene by *Pseudomonas chlororaphis* strain 23aP. The derivatives and several possible intermediates, which have not been detected by GC-MS or HPTLC analyses, are shown in brackets. Compounds: P1, 1,2-phenanthrenedihydrodiol; P2, 1,2-dihydroxyphenanthrene; P3, 3-(1-naphthyl)allyl alcohol; P4, *cis*-2-oxo-4-(2′-hydroxynaphthyl)-but-3-enoic acid; P5, 2-hydroxy-1-naphthoic acid; P6, naphthalene-1,2-diol; P7, 2-hydroxybenzalpyruvic acid; P8, 3,4-phenanthrenedihydrodiol; P9, 3,4-dihydroxyphenanthrene; P10, *cis*-2-oxo-4-(1′-hydroxynaphthyl)-but-3-enoic acid; P11, 1-hydroxy-2-naphthoic acid; P12, α-naphthol; P13, 4-hydroxy-1-tetralone; P14, 9,10-phenanthrenequinone; P15, 9*H*-fluorene-9-one; P16, diphenic acid; P17, 2-phenylbenzaldehyde; P18, phthalic acid. Abbreviations: TCA—tricarboxylic acid cycle, *—non enzymatic oxidation. Compounds P3, P7, P12, P14, P15, P17, and P18 (as alkylated derivatives) were identified by GC-MS based on the match of mass spectra (fragmentation and peak intensity, Table 1) with those from NIST (National Institute of Standards and Technology) mass spectra library and those reported in literature (details are available in the main text), while PHE was by comparison with authentic standard.

Since the utilization of PHE by bacteria is a multistep sequential transformation process and microorganisms display diverse catabolic activities, to assess the potential of hydrocarbon degradation, it is necessary to identify intermediate and end metabolic products. The combined data from the GC-MS analyses of exometabolites accumulated during the growth of *Pseudomonas chlororaphis* strain 23aP in the presence of phenanthrene in optimized conditions allowed us to identify *o*-phthalic acid, 3-(1-naphthyl)allyl alcohol, 2-hydroxybenzalpyruvic acid, *α*-naphthol, and 2-phenylbenzaldehyde. The HPTLC analysis revealed a compound that was not detected in GC-MS, with fluorescent characteristics and a migration coefficient similar to that of 1H2NA according to published data [25,26]. Considering the intermediate metabolites identified, the degradation pathway of PHE by strain 23aP was proposed (Figure 3). The recognition of 3-(1-naphthyl)allyl alcohol (P3) might indicate that the initial dioxygenation occurred at phenanthrene C1,2-positions with subsequent extradiol ring-cleavage and decarboxylation [43]. The same compound was confirmed among products of PHE degradation by *Aeromonas salmonicida* subsp. *achromogenes* strain NY4 [43] and *Stenotrophomonas maltophilia* strain JPHC3Z2B [24]. The occurrence of *α*-naphthol (P12), i.e., a product of 1H2N (P11) oxidation, proved the 3,4-*cis*-diol (P9) pathway. The lower *α*-naphthol route is an alternative to the much more common ring opening in 1H2N by both *ortho*- and *meta*-cleavage and can lead to both salicylic acid and phthalic acid production, but more often the former. Phenanthrene biodegradation via salicylate was confirmed for some strains of *Pseudomonas* [20,25,26] and *A. salmonicida* [43] via salicylate and *Rhizobium* [23] via phthalate. In the case of strain 23aP, the occurrence of alkylated derivatives of *o*-phthalic acid and no trace salicylic acid in the culture fluids suggests that *α*-naphthol (P12) is exclusively metabolized to phthalates (P18).

Another commonly encountered metabolite in the PHE degradation process is 2-hydroxybenzalpyruvic acid (P7) [24], which was detected in our GC-MS analyses. This lower pathway metabolite of 1,2- and 3,4-pathways is created as a result of decarboxylation and hydroxylation of 2H1H (P6) or 1H2N (P11) to produce naphthalene-1,2-diol (P6), which is further degraded through *meta*-cleavage [36,38]. 2-hydroxybenzalpyruvic acid is then transformed to salicylate [36]. This pathway was identified during PHE degradation by *Pseudomonas aureofciens* [44], *S. maltophilia* strain C6 [37], or *Sphingobium* sp. strain PNB [35]. However, no trace salicylic acid was detected in the strain 23aP culture medium in the current investigations, as in the case of the *S. maltophilia* strain JPHC3Z2B [24]. An explanation for this phenomenon may be found in an earlier report postulating that the appearance of particular products of PHE degradation in the medium is caused by the difference between the rates of exometabolite formation and its involvement in the next biochemical stage [44]. Thus, it is possible that the accumulation of salicylic acid would have occurred during prolonged incubation of 23aP with PHE, as in the case of *S. maltophilia* strain C6 [37]. However, to fully settle the pathway of PHE degradation by strain 23aP, the genome should be inspected in search of genes of the upper and lower pathways.

Lastly, among ethyl-acetate-extractable metabolites, the GC-MS chromatograms registered compounds generated after the initial oxygenation of PHE in the “K region”, e.g., 9,10-phenanthrenequinone and 9*H-*fluorene-9-one. Since they were also present in the abiotic controls, they may have been generated through non-enzymatic oxidation. According to previous reports, these PHE derivatives are common in ambient particles where they could be formed via the attack of free radicals [33]. However, the identification of 2-phenylbenzaldehyde (P17) in the culture fluids, which was absent in the abiotic controls, may indicate that strain 23aP is able to further metabolize these intermediate products. Dioxygenation of phenanthrene in the C9,10- position is usually a major pathway in Gram-positive bacteria but also in some Gram-negative bacteria, e.g., *S. maltophilia* strain C6 [37] and some *Pseudomonas* [22]. Usually, 2,2′-diphenic acid is the end product of this route [11]. Sometimes, it can be metabolized to biphenyl-2-carboxylic acid followed by gradual transformation to phthalic acid, as in the case of *Mycobacterium* [36,45]. Moreover, bacteria capable of utilizing fluorene, such as strain 23aP, can transform 9*H*-fluorene-9-one into 2′-carboxy-2,3-dihydroxybiphenyl [11], which is closely related to 2-phenylbenzaldehyde. Thus, the use of 23aP in consortia containing biopreparations for soil decontamination could help to improve the process and the further biotransformation of intermediate products of PHE degradation into simpler compounds included in the TCA cycle.

The biodegradation of aromatic pollutants with the participation of bacteria occurs in cytoplasm; however, high hydrophobicity, low solubility, and adsorption into the soil matrix are the limiting factors of their bioavailability and removal from the environment. Therefore, chemically different biosurfactant molecules produced by microorganisms and reducing surface tension or interfacial tension, changing the microstructures and functional groups of bacterial cell envelopes, and enhancing the cell membrane potential and surface cell hydrophobicity can increase the bioavailability and utilization of PAHs for microorganisms. *Pseudomonas* strains predominantly produce glycolipid biosurfactants called rhamnolipids [46]. In the current study, we have confirmed that strain 23aP can synthesize extracellular rhamnolipids and, in the presence of PHE and co-metabolites (Glc or tryptone), significantly increase its cell surface hydrophobicity in comparison with the PHE-alone variant. As reported by Ma et al. [29], increased hydrophobicity of the cell surface of Gram-negative bacteria is related to the substitution of lipopolysaccharide molecules with rhamnolipids in the outer membrane. In the case of 23aP, the data obtained indicated the production of cell-associated RhaLs in the presence of both co-metabolites. Both glucose and tryptone supported the fast growth of strain 23aP, and quorum sensing (QS) is known to regulate the production of rhamnolipids in *Pseudomonas* [47], which may additionally confirm the conclusions formulated in the present study.

To summarize, bacteria of the *Pseudomonas* genus are known, among other things, for their ability to decompose a wide range of substrates, including aliphatic and aromatic compounds [48], and some of them additionally possess the ability to promote plant growth and development [49]. Our preliminary research has shown that, in addition to the ability to degrade phenanthrene and produce biosurfactants, the studied *P. chlororaphis* 23aP strain has mechanisms related to the promotion of plant growth. Thus, this strain can be used in the bioaugmentation process, one of the biological methods supporting the removal of pollutants from a contaminated environment. Introduction of the 23aP strain into the soil in industrial areas, e.g., fuel reloading stations or the soil of agricultural areas adjacent to expressways, can increase and support the pool of indigenous microorganisms specialized in removing pollutants, which will be more effective and significantly accelerate the process of cleaning up and restoring contaminated areas. It is worth noting that strains used in bioaugmentation cannot be pathogens towards the native microbiota. They should not produce toxins but should be resistant to environmental changes and effective in removing contaminants. Another desirable feature of strains used in the bioaugmentation process is the ability to synthesize polysaccharide substances that facilitate aggregation, adhesion, and biofilm formation. Therefore, further research in laboratory conditions on the use of the 23aP strain in bioaugmentation is needed.

## 4. Materials and Methods

### 4.1. Bacterial Strain Isolation and Media

The study was conducted on the strain of *Pseudomonas chlororaphis* 23aP (23aP), which was originally isolated from root nodules of *Chamaecytisus albus* (Hacq.) Rothm., i.e., a plant growing in the southeastern part of Poland, with a standard method described by Wdowiak-Wróbel et al. [50]. The roots of *Ch. albus* were surface-disinfected by sequential washing with sterile water, 0.1% HgCl_2_ (*w*/*v*) for 1 min, 95% ethanol (*v*/*v*) for 1 min, and lastly several times with sterile water to remove epiphytes. The suspension obtained from crushed nodules [51] was streaked on yeast–mannitol medium (YEM) and incubated at 28 °C for 3–5 days. Cultivable isolates were purified by streaking several times on YEM agar. Pure cultures were stored at −20 °C in YEM medium with 50% (*v*/*v*) glycerol.

PHE-degrading isolates were selected by enrichment of the mineral salt agar medium with sprayed phenanthrene as the sole source of carbon and energy, as previously described [52]. Isolates that developed clear zones around colonies after the incubation in dark were considered PHE-degrading bacteria. Next, selected PAH-utilizers were tested for their tolerance and acclimatization to high levels of PAHs. For this purpose, isolates from the MSM medium with glucose as a carbon source (0.1% Glc) were inoculated in an equal quantity into the MSM medium enriched with phenanthrene (25 ppm). Every 7 days, bacterial pellets obtained from whole cultures were transferred into fresh MSM with increasing PHE content (50, 100, 200, and 500 ppm). CFUs were determined at each time point. Of the four strains tested, the one that showed the highest CFU at the end of the study was chosen for further research. It was also tested for utilization of other PAHs (fluorene, acenaphthene, and anthracene) on MSM agar media with sprayed hydrocarbons.

Between the experiments, the selected *Pseudomonas chlororaphis* 23aP strain was stored on MSM agar enriched with PHE to maintain selective pressure and subcultured weekly. The 23aP strain was deposited in the Bank collection of the Department of Genetics and Microbiology, Maria Curie-Skłodowska University in Lublin, Poland.

The basic MSM medium contained 1.73 g/L of K_2_HPO_4_, 0.68 g/L of KH_2_PO_4_, 0.1 g/L of MgSO_4_ × 7 H_2_O, 1 g/L of NH_4_NO_3_, 1.0 g/L of NaCl, 1 mL of trace salt solution, and 1 L of milliQ water. The pH of the medium was initially adjusted to 6.8 with 1.0 M HCl before sterilization. The trace salt solution was composed of 20 mg of CaCl_2_, 30 mg of FeCl_3_, 0.5 mg of CuSO_4_, 0.5 mg of MnSO_4_ × H_2_O, and 10 mg of ZnSO_4_ × 7 H_2_O per 1 L of milliQ water.

The stock solution of PHE dissolved in acetone was used (1250 ppm) for the preparation of MSM broth medium enriched with phenanthrene. Before the experiments, an appropriate volume of the PHE solution was poured into sterilized tubes/flasks and, after evaporation of acetone with a N_2_ stream, an appropriate volume of MSM medium was added, followed by sterilization by autoclaving at 121 °C for 20 min.

### 4.2. Phylogenetic Analysis of Selected Strain

The isolation of DNA from strain 23aP was carried out using a method described by Pitcher et al. [53]. The purity and concentration of genomic DNA were checked by NanoDrop™ 2000/2000c measurements (Thermo Fisher Scientific, Wilmington, DE, USA). The extracted DNA was stored at −20 °C.

The phylogenetic analysis of the isolate was determined using multilocus sequence analysis (MLSA), i.e., concatenating the sequences of *gyrB*, *rpoB*, and *recA* genes. These sequences were amplified using primer pairs described in previous studies [54,55]. All analyzed sequences were amplified in the PCR reaction using a Taq PCR Master Mix (2x) kit (EURx, Gdansk, Poland) according to the manufacturer’s instruction. A total 50 ng of template DNA and 0.4 mM of forward and reverse primers were added to the reaction mixture. PCR amplifications were performed using a thermal cycler (TProfessional BASIC 96 Gradient, Biometra GmBH, Göttingen, Germany) in the following conditions: initial denaturation at 94 °C for 2 min, followed by 35 cycles of denaturation at 94 °C for 30 s, annealing at 54 °C for 20 s, and extension at 72 °C for 2 min, with a final extension step of at 72 °C for 7 min.

The amplicons were purified using a Clean-Up purification kit (A&A Biotechnology, Gdansk, Poland) and sequenced with a BigDye^TM^ Terminator Cycle sequencing kit (Applied Biosystems, Foster City, CA, USA). The reactions were read using a 3500 Genetic Analyzer according to the procedure specified by the producer (Applied Biosystems, Foster City, CA, USA). The sequences were compared with relevant sequences in the GenBank database using the BLAST tool. The ClustalX2.1 program was used for sequence alignments analyzed in the GeneDoc program [56]. A phylogenetic tree was constructed based on the generated sequence set using the MEGA11 program with the Neighbor-Joining (NJ) method [57]. The two-parameter Kimura model was used as a nucleotide substitution model. The jModelTest selected the best-fitting evolutionary model for each tested gene [58]. The statistical significance of the tree was evaluated with the bootstrap test (1000 replicates). The phylogenetic tree was represented in the TreeView program [59].

### 4.3. Plant-Growth-Promoting Properties

The plant-growth-promoting mechanisms of strain 23aP were studied. The siderophore, hydrogen cyanide (HCN), and indole-3-acetic acid (IAA) production assays were carried out [60]. IAA production was determined according to Tiwari et al. [61]. The ability to solubilize phosphates was tested on an insoluble medium containing phosphates (tricalcium phosphate medium, TCP) based on the formation of transparent zones around bacterial colonies (positive reaction) [62]. The cellulolytic activity of the tested strain was determined on modified Congo red cellulose agar according to the previously described method [62,63]. The presence of a lightened zone around the colony indicated a cellulose hydrolyzing effect. To check the ability of the 23aP strain to carry out proteolysis, the proteolytic activity on a milk agar plate was performed according the procedure described by Morandi et al. [64]. The production of HCN was determined according to the method described by Lorck [60].

### 4.4. Optimization Parameters for Phenanthrene Degradation by the DCPIP Assay

Since the growth of bacteria depends on various environmental factors that may affect the effectiveness of PHE biodegradation, the culture of strain 23aP was optimized in relation to selected physicochemical parameters in single-component experiments. The degree of PHE degradation was evaluated with the colorimetric method using 2,6-dichlorophenol indophenol (DCPIP) indicator. The electron acceptor stock solution was prepared as follows: DCPIP (Sigma) was dissolved in a small amount of ethanol over low heat and then adjusted to the desired concentration (7 mM) with sterile reverse osmosis water. To prepare for the inoculation, bacteria were scraped from the PHE-enriched MSM agar plates followed by suspension in sterile MSM to their initial density OD_600_ = 0.75. The bacteria were then washed twice in fresh MSM by centrifugation and, finally, suspended in an initial portion of MSM broth medium.

The reaction mixtures for the phenanthrene biodegradation studies contained 4 mL of liquid MSM supplemented with 100 mg/L of PHE (except when otherwise stated), to which 40 μL of a sterile stock solution of DCPIP and 40 μL aliquots of the bacterial suspension for inoculation were added, just before the experiments. The incubation was carried out in the dark at 28 °C with agitation at 140 RPM (except when otherwise stated) for 4 days.

The PHE substrate utilization was estimated based on the degree of DCPIP decolorization. At tested time points, 500 μL of the reaction mixtures were taken from each sample and transferred to Eppendorf tubes. Then, cellular debris and remains of undissolved PHE crystals were pelleted by centrifugation (14,500 RPM/10 min at room temperature) and the reaction mixture (200 μL per well in duplicate) was transferred to a 96-well titration plate. The *A*_600_ absorbance measurements were made using a microplate reader (Asys UVM 340, ASYS Hitech GmbH, Eugendorf, Austria) at time t0 (*At*0) and at 1 day intervals (*Atx*). The percentage of indicator decolorization was calculated using the formula given below. Media that were not inoculated with bacteria were used as controls of abiotic PHE degradation.

$$\frac{At0-Atx}{At0}\times 100\%$$

The effects of nitrogen sources (0.1% ammonium nitrate, 0.1% tryptone), the initial concentration of PHE (6, 12.5, 25, 50, and 100 ppm), and the rate of agitation (140 RPM versus stationary cultures) were tested. Batch experiments for classical optimization were carried out by keeping all variables constant except one, whose optimized value needed to be found. The optimized value for each variable was selected and kept constant in further experiments.

### 4.5. Identification of Metabolites of PHE Degradation

To obtain phenanthrene degradation metabolites, cultures of strain 23aP were prepared in 100 mL flasks containing liquid MSM (50 mL) enriched with PHE (50 ppm) and NH_4_NO_3_ (2.5 g/L). The flasks were incubated in the dark in static conditions at 28 °C for 2 and 7 days. After the incubation periods, cellular debris were pelleted by centrifugation (9500× *g*/20 min at 4 °C, centrifuge Sigma 6-16KS, Sigma, Osterode am Harz, Germany), followed by filtration using PTFE syringe filters (0.22 μm, Alfatech Technology, Poznan, Poland). The cell-free supernatants were triple-extracted with ethyl acetate (1:1, *v*/*v*). The aqueous phases were acidified to pH 2.5 with 6M HCl and extracted in the same manner. Neutral and acidic organic extracts combined for samples from the different time points were dried over anhydrous sodium sulfate and evaporated to dryness in vacuo at 40 °C. The residues were redissolved in 2 mL of chloroform and triple-washed with deionized water (1:1, *v*/*v*). The organic layers were dried on anhydrous sodium-sulfate-packed columns and chloroform was removed in a nitrogen stream. Control experiments were performed without bacterial cells according to the same procedures.

Metabolic intermediates resulting from the degradation of PHE were identified: (i) without derivatization; (ii) as their methyl esters (2M methanolic HCl prepared from acetyl chloride and methanol/80 °C/2 h); and, subsequently, (iii) trimethylsilylated (HMDS/TMCS/pyridine, 3:1:9, *v*/*v*/*v*; Sigma-Aldrich, St. Louis, MO, USA). The samples were analyzed using a gas chromatograph (Agilent Technologies, Santa Clara, CA, USA, instrument 7890A) coupled with a mass selective detector (Agilent Technologies, Santa Clara, CA, USA, instrument MSD 5975C, inert XL EI/CI). The GC-MS system was equipped with an HP-5MS capillary column (30 m × 0.25 mm) with helium as a carrier gas (1 mL × min^−^^1^). The analyses were carried out using the EI mode (70 eV) with the following temperature program: 150 °C (5 min), then up to 310 °C with an increase of 5 °C per minute, and 10 min at 310 °C.

### 4.6. Hydrophobicity Test—Microbial Adhesion to Hydrocarbons (MATH)

The hydrophobicity of the bacteria was tested using the MATH method, as described previously [30], with minor modifications. The change in the surface properties of cells in response to changes in the culture conditions was evaluated. Variants of MSM cultures enriched with PHE (50 ppm) and additionally with glucose (0.1%) or tryptone (0.1%) were tested. Bacteria obtained from appropriate cultures were pelleted by centrifugation (14,500 RPM/10 min; Eppendorf MiniSpin plus, Eppendorf, Germany) and then suspended in PUM buffer (22.2 g K_2_HPO_4_ × H_2_O, 7.26 g KH_2_PO_4_, 1.8 g urea, 0.2 g MgSO_4_ × 7H_2_O, and 1 L of milliQ water) to an optical density of about 0.5 (OD_1_) at 405 nm. Total 200 µL aliquots of bacterial suspensions were supplemented with 100 µL of dodecane, left for 10 min at room temperature, and vortexed exhaustively for 120 s. After 15-min equilibration, the optical density of the lower phase was measured (OD_a_) on a microplate reader (Asys UVM 340, ASYS Hitech GmbH, Eugendorf, Austria). The degree of hydrophobicity was calculated as follows:

% hydrophobicity = 100 − 100(OD_a_/OD_1_**)**

### 4.7. Biosurfactant Production

For biosurfactant production, 100 mL of MSM amended with glucose (0.1%) as a sole carbon substrate and tryptone (0.1%) as a nitrogen source was inoculated with the seed culture (5% inoculum) and incubated with agitation at 140 RPM, at 28 °C, for 7 days. Before the extraction of the biosurfactant, a cell-free supernatant was obtained through centrifugation of culture broth for 20 min at 9500× *g* at 4 °C followed by filtration with a 0.2 μm syringe filter. Then, the supernatant was acidified to pH 2 using 6 N HCl and stored at 4 °C overnight. The material was divided in half and different extraction methods were applied to each portion to obtain as many biosurfactant homologs as possible. One half portion of the sample was treated according to the Folch extraction procedure by adding a CHCl_3_:MeOH mixture (2:1, *v*/*v*) to the supernatant sample to achieve a final CHCl_3_:MeOH:H_2_O ratio of 8:4:3 (*v*/*v*/*v*). The second portion of the cell-free supernatant was triple extracted with ethyl acetate (1:1, *v*/*v*). After vigorous agitation, the mixture was left stationary for phase separation. The collected chloroform and ethyl acetate layers were evaporated to dryness on a rotary evaporator at 40 °C under reduced pressure. The obtained samples were de-salted by extraction in the CHCl_3_:H_2_O (1:1, *v*/*v*) system and washing twice successively with water. The organic layers were dried on anhydrous sodium-sulfate-packed columns and chloroform was removed in the nitrogen stream. The crude biosurfactant preparations were suspended in the CHCl_3_:H_2_O (9:1, *v*/*v*) mixture to obtain a final concentration of 4 mg/mL. They were applied to HPTLC chromatography and ESI-MS analyses.

### 4.8. HPTLC Analysis

Intermediate metabolites of the phenanthrene pathway were resolved on 10 × 10 cm HPTLC silica gel 60 F_254_ plates (Merck, Darmstadt, Germany) using hexan:chloroform:acetic acid (10:3:2, *v*/*v*/*v*) as a solvent system. The metabolites were identified by comparing R_f_ and UV-fluorescence properties at wavelengths of 365 nm [25]. HPTLC of crude rhamnolipid preparations was performed with a carrier solution of CHCl_3_:MeOH:H_2_O, 65:15:2 by volume [65]. Visualization was made with iodine vapor. The same system was also applied to analyze PHE metabolites.

### 4.9. ESI-MS Analysis

Before the analysis, samples of crude biosurfactants were diluted with a mixture containing 2-propanol:acetonitrile:water (2:1:1, *v*/*v*/*v*) to obtain a final concentration of 1 μg/μL. The sample was then filtered using a PTFE syringe filter (0.22 μm, Alfatech Technology, Poznan, Poland) and transferred to a new glass vial. The sample was injected by infusion into the ion source at a flow rate of 10 μL/min, and MS spectra were recorded for 5 min.

ESI-MS spectrometry was performed using a SYNAPT G2-*Si* HDMS instrument (Waters Corporation, Milford, MA, USA) operating in negative electrospray mode. Data acquisition was performed in the range of 100–900 *m*/*z* using MassLynx version 4.1 software (Waters Corporation, Wilmslow, UK). The spectrometer conditions were as follows: capillary voltage 3.00 kV, sampling cone 60 V, and source offset 80 V. The ion source temperature was set at 120 °C and the desolvation temperature was 250 °C. The cone gas flow and the desolvation gas flow were set to 100 L/h and 600 L/h, respectively. For the MS2 experiments, isolated precursor ions were fragmented at an ion trap, with the voltage set at 45 V. Data were collected for 120 s for each selected precursor ion. The mass spectrometer was calibrated against sodium iodide (Sigma-Aldrich, St. Louis, MO, USA).

### 4.10. Statistical Analysis

All experiments were performed independently three times, and each experiment comprised two samples. The values obtained were subjected to statistical analyses performed with GraphPad Prism 6.0 software and presented as means (±SD) of three independent experiments. The significance level of *p* < 0.05 was established using one-way analysis of variance (ANOVA) and Tukey’s test.

### 4.11. Accession Numbers

The GenBank accession numbers for the sequences determined in this work are OR568610, OR568611, and OR568612. The accession numbers of the reference strains are given on the phylogram.

## 5. Conclusions

In summary, *Pseudomonas chlororaphis* strain 23aP obtained from the nodules of *Ch. albus* seems to have the potential to be used in PHE bioremediation, particularly with the microbial-assisted phytoremediation technique, for the following reasons: (i) it utilizes phenanthrene as the only source of carbon and energy and transforms it into compounds directed to the tricarboxylic acid cycle; (ii) it remains viable in the presence of high PHE concentrations (up to 500 ppm); (iii) it produces rhamnolipids and, in the presence of PHE, changes the properties of the cell surface to become more hydrophobic, which may be reflected by an increased bioavailability of phenanthrene and, thus, increased efficiency of its biodegradation; and (iv) it has some PGP characteristics.

## Figures and Tables

**Table 1:** GC-MS spectral data of metabolites from the degradation of phenanthrene by Pseudomonas chlororaphis strain 23aP.

Retention Time (min)	*m/z* of Major ion Peaks (Ion Type, % Relative Abundance)	Suggested Structure ^1^
5.580	208 (M^+^, 32), 193 (100),151 (16), 73 (32)	Acetophenone ^2^
6.353	144 (M^+^, 100), 115 (4), 116 (72), 89 (10)	*α*-naphthol (P12)
7.039	278 (M^+^, 16), 263 (100), 247 (1), 191 (3), 175 (5), 147 (2)	2-hydroxybenzalpyruvic acid ^2^ (P7)
7.517	216 (M^+^, 81), 201 (100), 185 (16), 145 (31), 141 (17), 127 (18), 115 (14)	*α*-naphthol ^2^ (P12)
7.600	171 (29), 152 (98), 84 (35), 83 (45), 74 (100), 55 (81)	nonanedioic acid, monomethyl ester
8.037	177 (27), 166 (16), 149 (100), 83 (27), 71 (27), 57 (36)	diethyl phthalate (P18)
11.219	180 (M^+^, 100), 152 (37), 151 (22), 76 (16)	9*H*-fluorene-9-one (P15)
11.551	184 (M^+^, 100), 152 (11), 139 (15), 92 (8)	3-(1-naphthyl)allyl alcohol (P3)
12.321	178 (M^+^, 100), 176 (18), 152 (9), 151 (7), 89 (13), 76 (16)	9-methylene-9*H*-fluorene
13.860	223 (8), 149 (100), 104 (1), 57 (18)	phthalic acid, bis-isobutyl ester (P18)
13.943	181 (100), 167 (25), 152 (31), 76 (9)	2-phenylbenzaldehyde (P17)
15.752	278 (M^+^, 0.5), 223 (5), 149 (100)	dibutyl phthalate (P18)
15.960	208 (M^+^, 18), 180 (100), 152 (79), 151 (46), 76 (40)	9,10-phenanthrenequinone (P14)
25.879	279 (11), 167 (34), 149 (100), 71 (18), 57 (23)	di-*n*-octyl phthalate (P18)

^1^ Identification was based on the match of mass spectra (fragmentation and peak intensity) with those from NIST (National Institute of Standards and Technology) mass spectra library and those reported in literature (details are available in the main text). ^2^ Trimethylsilylated methyl ester derivatives. M^+^ molecular ion. P3, P7, P12, P14, P15, P17, P18—metabolite designations identical to Figure 3 (Discussion and in Figure 3 caption).

**Table 2:** Homologs of rhamnolipids produced by *Pseudomonas chlororaphis* strain 23aP assigned on the basis of data recorded with ESI-MS/MS2 operating in negative ionization mode.

Congener	Formula	Obs *m/z* [M-H]^−^	Calc *m/z* [M-H]^−^	Fragment Diagnostic Ions (*m/z*)
^2^Rha-C_8_-C_8_	C_22_H_39_O_9_	447.4407	447.2594	nd
^2^Rha-C_10_-C_8:1_	C_24_H_41_O_9_	473.3032	473.2750	333.1136 (Rha-C_10_)
^1^Rha-C_12:2_-C_10_	C_28_H_47_O_9_	527.2681	527.3220	357.1940 (Rha-C_12:2_)
^1^Rha- C_12:1_-C_12:1_	C_30_H_51_O_9_	555.3016	555.3533	195.0625 (C_12:1_)
^1^Rha-Rha-C_8_-C_10_	C_30_H_53_O_13_	621.3422	621.3486	311.1824 (C_8_-C_10_), 339.2098 (Rha-Rha), 451.2752 (Rha-Rha-C_8_)

Abbreviations: Obs—molecular mass recorded; Calc—molecular mass calculated; nd—not detected; Rha—rhamnose; 1—sample extracted with Folch method; 2—sample extracted with ethyl acetate.

## Data Availability

The data presented in this study are available on request from the corresponding author. The data are not publicly available due to privacy.

## Supplementary Materials

The following supporting information can be downloaded at: https://www.mdpi.com/article/10.3390/molecules28227581/s1, Figure S1. *Pseudomonas chlororaphis* strain 23aP. (a,b) Colony morphology on 79CA agar medium; (c) the image of Gram staining of bacteria cultured on 79CA agar medium; (d,e) colony morphology on the phenanthrene-coated MSM agar plate—the insert in D reveals clearance zones of PHE crystals around the colonies; (f) the image of Gram staining of bacteria cultured on the phenanthrene-coated MSM agar. Table S1. Plant-growth-promoting properties of *P. chlororaphis* 23aP strain. Figure S2. HPTLC analysis of ethyl-acetate-extractable metabolites of PHE degradation by strain 23aP. Commercial standards: 1. vanillic acid, 2. veratric acid, 3. ferulic acid, 4. salicylic acid, 5. α-naphthol, 7. Phenanthrene. Samples: 6. 2-day abiotic control, 8. 7-day abiotic control, 9. 2-day culture fluid, 10. 7-day culture fluid. Solvent system used in the study: hexan:chloroform:acetic acid (15:4.5:3, by volume). Chromatograms visualized in (a) UV wavelength at 365 nm; (b) UV wavelength at 254 nm. Table S2. Data from HPTLC in solvent system hexan:chloroform:acetic acid (15:4.5:3 by volume). Figure S3. HPTLC analysis of ethyl-acetate-extractable metabolites of PHE degradation by strain 23aP. Commercial standards: 1. vanillic acid, 3. ferulic acid, 4. salicylic acid, 5. α-naphthol, 7. Phenanthrene. Samples: 10. 7-day culture fluid. Solvent system used in the study: chloroform:methanol:water (65:15:2, by volume). Chromatograms visualized in (a) UV wavelength at 365 nm, (b) UV wavelength at 254 nm, and (c) iodine vapor. Table S3. Data from HPTLC in solvent system chloroform:methanol:water (65:15:2, by volume). Figure S4. Thin-layer chromatography (HPTLC) analysis of crude rhamnolipids obtained from broth cultures of strain 23aP on MSM medium supplemented with trypton (0.1%) and Glc (0.1%). Sample 1 (1) material from Folch extraction. Sample 2 (2) material extracted with ethyl acetate. Developed mixture contained chloroform:methanol:water (65:16:2; by volume). Chromatograms were developed with iodine vapor.
