# Supplementary material for: Assessment of Phenanthrene Degradation Potential by Plant-Growth-Promoting Endophytic Strain Pseudomonas chlororaphis 23aP Isolated from Chamaecytisus albus (Hacq.) Rothm
Source: Molecules. 2023 Nov 14;28(22):7581. doi: 10.3390/molecules28227581 (PMC10673423; doi:10.3390/molecules28227581)
Supplement: Supplementary file 1 [file molecules-28-07581-s001.zip › molecules-2667208-supplementary.pdf]

Assessment of phenanthrene degradation potential by plant growth-promoting endophytic strain *Pseudomonas chlororaphis* 23aP isolated from *Chamaecytisus albus* (Hacq.) Rothm.

Magdalena Anna Karaś*, Sylwia Wdowiak-Wróbel, Monika Marek-Kozaczuk, Wojciech Sokołowski, Krystsina Melianchuk, and Iwona Komaniecka *

Department of Genetics and Microbiology, Institute of Biological Sciences, Faculty of Biology and Biotechnology, Maria Curie-Skłodowska University, Akademicka 19, 20-033 Lublin, Poland
Correspondence: magdalena.karas@mail.umcs.pl (M.A.K) and iwona.komaniecka@mail.umcs.pl (I.K.)

2.1. Isolation and identification of the PHE-degrading-strain with PGP activities

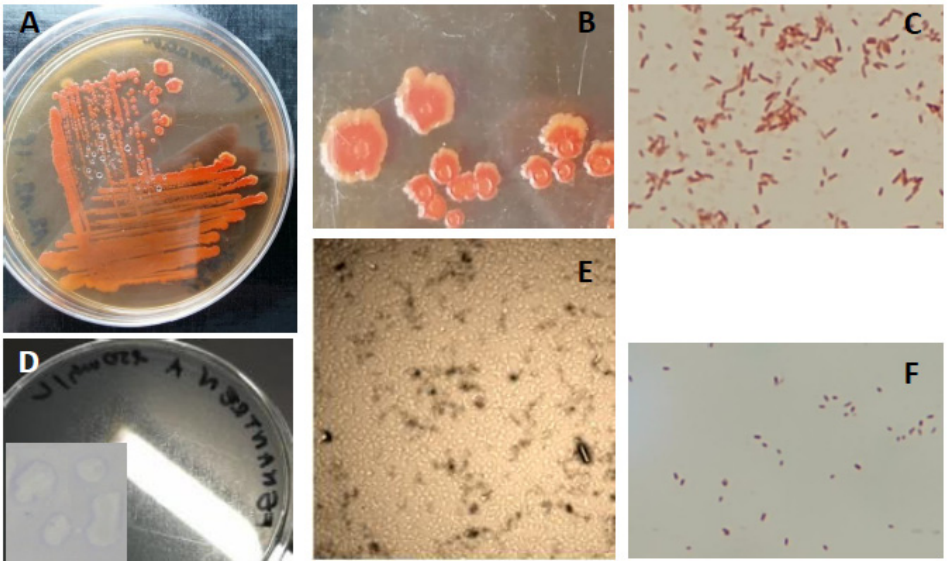

Figure S1. *Pseudomonas chlororaphis* strain 23aP. (a, b) Colony morphology on 79CA agar medium; (c) the image of Gram staining of bacteria cultured on 79CA agar medium; (d, e) Colony morphology on the phenanthrene-coated MSM agar plate; the insert in D reveals clearance zones of PHE crystals around the colonies; (f) the image of Gram staining of bacteria cultured on the phenanthrene-coated MSM agar.

Table S1. Plant growth promoting properties of *P. chlororaphis* 23aP strain

Strain	Siderophore production	HCN production	Phosphate solubilization	Cellulolytic activity	Proteolytic activity	(IAA) production
Pseudomonas chlororaphis aP23	+	+	+	+	+	-

+ present; - absent; HCN – hydrogen cyanide; IAA - indole-3-acetic acid

2.3. Phenanthrene metabolic intermediates

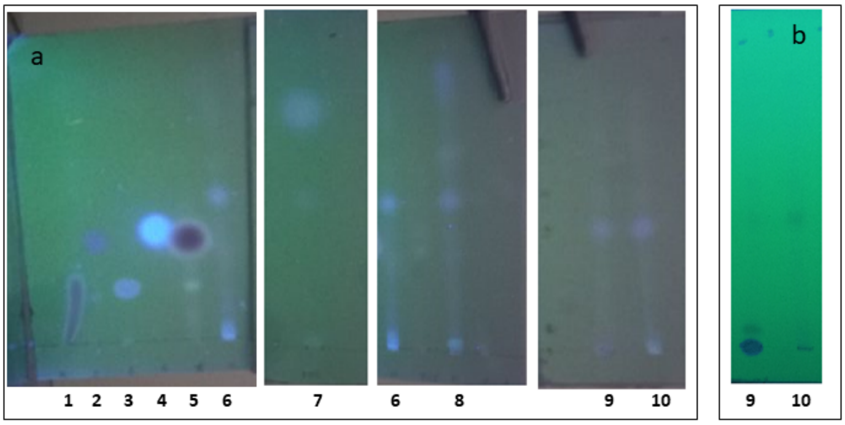

Figure S2. HPTLC analysis of ethylacetate-extractable metabolites of PHE degradation by strain 23aP. Commercial standards. 1. vanilic acid, 2. veratric acid, 3. ferulic acid, 4. salicylic acid, 5. α-naphthol, 7. phenanthrene; Samples: 6. 2-day-abiotic control, 8. 7-day-abiotic control, 9. 2-day-culture fluid, 10. 7-day-culture fluid. Solvent system used in the study: hexan:chloroform:acetic acid (15:4.5:3, by volume). Chromatograms visualized in (a) UV wave length at 345 nm; (b) UV wave length at 254 nm

Table S2. Data from HPTLC in solvent system hexan:chloroform:acetic acid (15:4.5:3, by volume).

**Compound**	**R_f**	**UV-fluorescence**
1. vanilic acid	0.19	Purple, non-fluorescent
2. veratric acid	0.35	Dark blue, non-fluorescent
3. ferulic acid	0.19	Blue, non-fluorescent
4. **salicylic acid**	0.36	Blue, fluorescent
5. **α-naphthol**	0.33	Purple, non-fluorescent
7. phenanthrene	0.71	Blue, non-fluorescent
6. 2-day-abiotic control	0.03; 0.46; 0.6	
8. 7-day-abiotic control	0.03; 0.46; 0.6; 0,66; 0,78	
9. 10. culture fluid	0.0; 0.07; **0.37**; 0.48; 0.63; **0.75**	

According to data obtained from GC-MS analyses, spots with R_f 0.48 (0.46) and R_f 0.63 (0.6) observed in HPTLC analysis can be attributed to 9*H*-fluorene-9-one and 9,10-phenanthrenequinone, respectively.

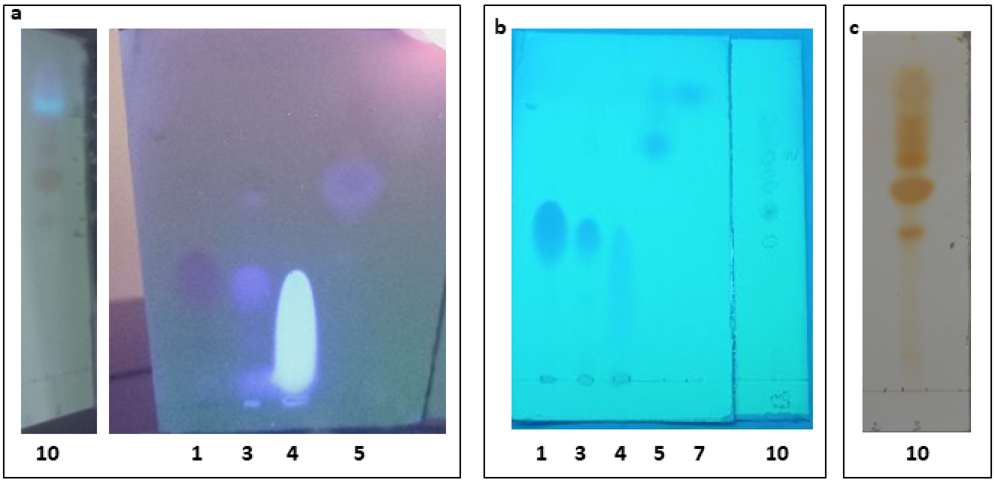

Figure S3. HPTLC analysis of ethylacetate-extractable metabolites of PHE degradation by strain 23aP. Commercial standards. 1. vanilic acid, 3. ferulic acid, 4. salicylic acid, 5. α-naphthol, 7. phenanthrene; Samples: 10. 7-day-culture fluid. Solvent system used in the study: chloroform:methanol:water (65:15:2, by volume). Chromatograms visualized in (a) UV wave length at 345 nm; (b) UV wave length at 254 nm; (c) iodine vapor

Table S3. Data from HPTLC in solvent system chloroform:methanol:water (65:15:2, by volume).

Compound	R_f
1. vanilic acid	0.47
3. ferulic acid	0.40
4. **salicylic acid**	0.39
5. **α-naphthol**	0.67
7. phenanthrene	0.82
10. culture fluid	0.09; 0.43; 0.59; **0.67**; 0.68; 0.77; 0.88

2.4. Mechanisms for increasing of phenanthrene bioavailability

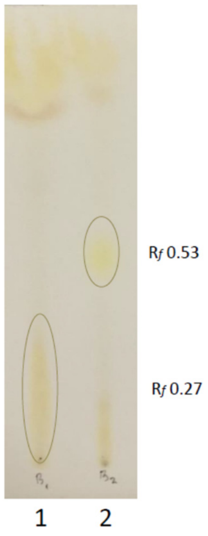

Figure S4. Thin layer chromatography (HPTLC) analysis of crude rhamnolipids obtained from broth cultures of strain 23aP on MSM medium supplemented with trypton (0.1%) and Glc (0.1%). Sample-1 (1) material from Folch extraction; Sample-2 (2) material extracted with ethyl acetate. Developing mixture contained chloroform:methanol:water (65:16:2; by volume). Chromatograms were developed with iodine vapor.
